# The Spatial Spillover Effect of Clean Energy Development on Economic Development: A Case of Theoretical and Empirical Analyses from China

**DOI:** 10.3390/ijerph20043144

**Published:** 2023-02-10

**Authors:** Minglin Wang, Si Tan, Yunzhe Wang, Zhengxia He, Shaolong Zeng

**Affiliations:** School of Economics, Hangzhou Normal University, Hangzhou 311121, China

**Keywords:** spatial spillover effect, clean energy development, economic growth, spatial Durbin model

## Abstract

Does clean energy development (CED) have a spatial spillover effect on economic growth (EG)? Using the panel data of 30 provincial administrative units from 2000 to 2019 in China, this study empirically investigates the spatial spillover effect of CED on EG. From the perspective of the supply side rather than the consumption side, using the spatial Durbin model (SDM), the study finds that CED does not have a significant impact on EG, while there is an apparent positive spillover effect of CED on EG in China, meaning that CED in one province can boost EG in the surrounding provinces. Theoretically, this paper provides a new perspective for studying the relationship between CED and EG. In practice, it provides a reference for further improving the government’s future energy policy.

## 1. Introduction

Although the current energy crisis in many countries has increased demand for fossil fuels in the short term, clean energy remains the key to solving humanity’s energy problems in the long term: fossil fuels are expected to decline from 80% of the global energy mix today to 75% by 2030 and around 60% by 2050 [1].

The development of energy saving, emission reduction, and the green and low-carbon economy is receiving increasing attention in all countries around the world. The French government has set a target of 40 percent of its electricity being generated from renewable sources by 2030, a ten-fold increase in its installed solar capacity by 2050 and the construction of 50 offshore wind farms [2]. Japan aims to increase the share of electricity generated from renewable sources to between 36 and 38 percent by 2030, according to the latest version of its Basic Energy Plan [3]. Egypt plans to install facilities to provide 43 gigawatts of solar power by 2035 in its Comprehensive Sustainable Energy Strategy 2035 [4]. 

China has continued to optimize its energy mix, with coal consumption falling to 56.8% between 2016 and 2020 [5]. China now ranks first in the world in a number of indicators, including the amount of installed hydropower, wind power, solar PV, and nuclear power facilities under construction. China has become a major player in driving global clean energy development by building the world’s largest clean power generation system. According to the Chinese government’s 14th Five-Year Plan for a Modern Energy System, the proportion of non-fossil energy consumption will increase to about 20 per cent and the proportion of non-fossil energy generation will reach about 39 per cent by 2025 [6]. In 2021, China launched the Action Plan for Carbon Capping in 2030 and will achieve carbon capping and carbon neutrality in an orderly manner [7].

Due to the vast size of China, there are huge differences across provinces in terms of resource endowment, industrial structure, technology level, and the stage of urbanization. Accordingly, there is considerable heterogeneity in the basis for developing the clean energy industry (CEI) in each province, resulting in an unbalanced CEI indifferent province. As building clean energy projects requires not only investing large amounts of capital and talent in research and development (R&D) but also constructing costly supporting infrastructure, these activities need to interact with each other across provinces. According to the first law of geography, two regions that are geographically close to each other are usually more interconnected than two regions that are further apart. The abbreviations of key terms showed in Table A1.

Therefore, it is worth investigating whether CED in one province can boost EG in the surrounding provinces. In other words, whether there is a spatial spillover effect and how strong it is. According to the first law of geography, two regions that are geographically close to each other are usually more connected than two regions that are further apart. Therefore, it is very interesting to investigate whether CED in one province can stimulate EG in the surrounding provinces, i.e., whether a spatial spillover effect exists.

While there is a small body of literature that has examined the spatial spillover of CED on EG, almost all of it is based on the consumption side of clean energy production (CEP). This research approach is appropriate for countries that are able to absorb clean energy products produced in the region. However, due to the mismatch between the production and consumption of clean energy in different regions, China has formed a CEI pattern dominated by western power of clean energy supply to the east, as local clean energy consumption in the western region is difficult. In addition, due to technical and market reasons, China has so far struggled to fully absorb its own clean energy power. This means that the consumption of clean energy is generally lower than the total CEP in China.

Therefore, it is necessary to study the spatial spillover of CED in China from the supply side. Theoretically, this paper provides a new perspective for studying the spatial spillover effect of CED on EG, and practically, it provides a reference for the Chinese government to further improve its policies to support CED in the future.

The structure of this paper is as follows. Section 2 provides a literature review and theoretical analysis of the impact of CED on EG and a hypothesis proposal. Section 3 is devoted to setting up the econometric model and data sources and describing the variables. Section 4 provides empirical results and robustness tests and an empirical analysis of the spatial effect of CED on EG. Conclusions and policy recommendations are presented in Section 5.

## 2. Literature Review and Theoretical Analysis

### 2.1. Literature Review

At present, studies on the relationship between CED and economic EG are relatively abundant in academic circles. Many scholars’ studies mainly focus on the causal relationship between CED and EG. As regards the causal relationship between CED and EG, there is still no consensus in the academic community [8,9,10,11,12,13,14,15].

First, some studies have found a one-way causal relationship between CED and EG. Pereira et al. (2021) conducted an empirical study using global panel data, and the results showed that CED’s influence on EG was negative or insignificant in the short term but might be positive in the long term [8]. In contrast, a study found a positive correlation between CED and EG in China [9]. Li et al. (2022) investigated the non-linear effects of CED, EG, and environmental pollution and found that there was an N-shaped curve relationship between EC and EG [10]. Hou et al. (2022) found the level of economic development increased in the early stage of CED; however, after the middle stage of CED, the level of economic development intensity decreased [11]. In other studies, there was no causal relationship found between CED and EG. Bao and Xu (2019) used bootstrap panel causality and found that there was no causal relationship between EC and EG, and they had no influence on each other [12].

Second, some studies have revealed the two-way causal relationship between CED and EG, that is, CED and EG influence each other and decide together, which represents the feedback hypothesis. Mohsin et al. (2021) applied the robust random effects method and Hausmann Taylor regression (HTR) to the panel data of 25 Asian developing countries from 2000 to 2016 and found that renewable energy consumption and EG were positively correlated in the short and long term with each other [13]. The same results have been found in studies of African countries such as Ghana [14]. One study supports the existence of a bidirectional causal relationship between CED and EG, using panel data of G7 countries for the period 1990–2011 [15].

Lastly, there is also some literature on the spatial spillover effect of CED on EG [16,17,18,19,20,21,22]. Marques et al. (2012) highlight the importance of considering a spatial spillover effect in the study of the relationship between CED and EG [16]. Magnaniet al. (2013) argue that administrative regions cannot be considered as independent individuals and that a simple panel model may be inappropriate. They found that renewable energy generation in Italy has positive spillover effects on the whole economy [17]. On the basis of Moran’s I and the spatial Durbin model, Bai et al. (2020) researched the spatial spillover of new energy development on CG of 21 developing areas in China from 2000 to 2017, and new energy was found to have a negative spatial spillover effect on the EG of neighboring areas [18].

Based on an analysis of existing studies, we believe that spatial spillover has not yet received sufficient attention from researchers, although the existence of spatial spillover has been identified in studies of overall energy or conventional energy [18,19]. Moreover, we find that, except for a very few scholars such as Magnani et al. (2013), most scholars (Inglesi-Lotz et al., 2015; Bao et al., 2019; Bai et al., 2020; Mohsin et al., 2021; Gyimah et al., 2021; Pereira et al., 2022; Li et al., 2022; Hou et al., 2022) use clean energy consumption as the core explanatory variable in their regression models when studying the relationship between CED and EG, rather than CEP.

However, due to technical and market reasons, not all CEP is consumed finally, then clean energy consumption is generally less than the total output of CEP, which makes it difficult to accurately measure CED. Therefore, there are significant limitations to using this variable as a core explanatory variable. Furthermore, in terms of the spatial spillover effect, CEP and associated clean energy technologies, projects, and expertise have more radiative pathways and are more powerfully radiative than clean energy consumption. For the above reasons, this paper applies a spatial effects model with CEP as the core explanatory variable to analyze the relationship between CED and EG to fill the gaps in existing research.

### 2.2. Theoretical Analysis of Spatial Spillover Effects

The current empirical research mainly examines the relationship between CED and EG through traditional econometric methods. The relationship between regions is not assumed to be independent of each other, and the spatial correlation between regions that may exist in CED is not considered. In fact, the level of CED in a region affects not only the EG of the region but also the EG of neighboring regions. According to the principle of spatial econometrics, traditional econometric methods may be biased when there are spatial effects. The EG of one region will also have an impact on the EG of the surrounding regions. This is generally referred to as the spatial spillover effect. EG itself also has a spatial spillover effect.

Investment has spillover effects on EG, which has been analyzed from externalities and spillover effects, meaning that it brings benefits not only to its own firms but also to other firms [23]. Romer proposed a knowledge spillover model, and he believed that the difference between knowledge and ordinary goods lies in the spillover effect of knowledge [24]. The concentration of an industry in a city promotes knowledge spillovers between firms, and thus the growth of that industry and that city [25]. Endogenous technological progress is the driving force behind EG. In the Romer model, the aggregate production function describes the relationship between the stock of capital, labor force, and creative technology and output. The human capital spillover model of Lucas pointed out that the spillover effect of human capital can be explained as learning from others or learning from each other [26]. A person with higher human capital will have a more favorable effect on the people around him/her and improve the productivity of the people around him/her. However, he/she will not benefit from it. Technology adoption, learning-by-doing, and EG were examined through a model in which firms choose which technologies to adopt as well as when to adopt them. In the adoptions, a company gains expertise in its technology which allows it to operate the technology more efficiently [27]. Subsequent studies by scholars have confirmed that there is a positive or negative spillover effect. However, due to the increase in regional distance, the spillover effect gradually decreases.

Therefore, in this paper, the spatial spillover effect of CED on EG refers to the fact that CED will not only have an impact on the EG of the province but will also affect the EG of other provinces. The spatial effect decreases as the distance increases. This spatial effect is mainly achieved in the following four ways.

#### 2.2.1. Spillover Effect of Science and Technology

Technology and knowledge are more fluid within a country than between countries, so they have a stronger spillover effect [28]. Some studies have found that technology spillovers within a country can promote its economic growth [29]. With the increase of CEP in the region, the technological level of the region will also increase. Industrial agglomeration is usually closely related to the agglomeration of technology and information, which brings sufficient innovation resources. Under the influence of the development of transportation and information technology, communication between regions will be strengthened, thus forming a network characteristic of regions. The most advanced science and technology will be extended to industries in the surrounding areas, rapidly raising the level of science and technology in the surrounding areas and continuing to promote the development of the regional economy.

#### 2.2.2. Demonstration Effect

While improving the EG in the region, CED projects can also set an example for other regions by giving full play to the benchmarking effect of savings and emissions reductions. The demonstration effect may force the surrounding governments to increase environmental regulation by increasing the environmental demands of residents in the region, thereby affecting the CED and EG. The demonstration effect can also promote the learning and follow-up of underdeveloped regions and achieve the purpose of promoting the EG of adjacent regions [30].

#### 2.2.3. Competition Effect

Clean energy is helped by government support for a high-tech and strategically emerging industry. In particular, as China’s economy enters an era of high-quality development, it has gradually become the general consensus of society to eliminate traditional energy, develop new energy, and take the path of the green, low-carbon, and circular economy. Some preferential and special policies must be provided by the central government to develop clean energy. This is also one of the usual international practices to support CED when CEI is not fully developed. However, because of China’s political tournament system for promoting local officials [31], there will be more competition between provinces than in other countries. Once a local government acquires the qualification to promote clean energy, it will receive some preferential and special policies from the higher government and the policies will not only bring a lot of capital, technology, and other production factors but it will also have negative spillover effects on the EG in the surrounding areas. When these preferential and special policies are implemented, there will be fierce competition among the provinces.

#### 2.2.4. Spatial Effect

Environmental problems are often diffused through the fluidity of the atmosphere and rivers. If the output of CEP in a certain area is low, the large amount of exhaust pollutants generated by the non-clean energy projects of enterprises in the area will be significantly higher, which will bring adverse effects on the economy of the surrounding areas. Conversely, increasing CEP and reducing pollutant emissions can also benefit the natural environment in adjacent areas. With the improvement of the natural environment, the mobility of the surrounding areas increases, the attractiveness of talents is enhanced, and the technological innovation ability is improved, which will have a positive impact on EG [32,33].

### 2.3. Propositional Hypothesis

According to the theoretical analysis of the spatial spillover effect, this paper proposes the hypothesis as below:

**Hypothesis** **1** **(H1):** *The impact of CED on EG has a spatial spillover effect*.

In summary, this paper intends to select 2000–2019 annual panel data of China’s 30 provinces and use the extended C-D production function to study the spatial spillover effects of CED on EG in China. In addition, this paper will put forward scientific and rational guidance and opinions for the development of China’s CEI and energy transformation. The 30 provinces include Anhui, Beijing, Fujian, Gansu, Guangdong, Guangxi, Guizhou, Hainan, Hebei, Henan, Heilongjiang, Hubei, Hunan, Jilin, Jiangsu, Jiangxi, Liaoning, Inner Mongolia, Ningxia, Qinghai, Shandong, Shanxi, Shaanxi, Shanghai, Sichuan, Tianjin, Xinjiang, Yunnan, Zhejiang, and Chongqing.

## 3. Establishment of Econometric Model and Variable Description

### 3.1. C-D Production Function and Its Improvement

EG, as a process in which the quantity of goods and labor produced by society continues to increase, is the result of the combined influence of many factors and is also constrained by many conditions. Most analyses of EG use the C-D production function. In the traditional C-D production function, the main factors affecting EG are technological progress, capital input, and human capital. We analyze these factors and then make improvements to the traditional C-D production function.

#### 3.1.1. Technical Progress

Technology and the development of the economy and society are closely related to each other [28,34,35]. If there is no investment in technological elements, there will be no increase in productivity factors, and no effective economic and social growth will be promoted. Technology is more permeable and mainly relies on three factors to promote economic and social growth. Technology has improved the human element of the basic elements of production and manufacturing, requiring workers to have contemporary scientific knowledge and technical skills, and the workers have gradually changed from simple manual workers to brain workers. At the same time, technology improves the factors of the basic elements of production, so as to promote the transformation and innovation of production technology and expand the depth and breadth of production labor objects. The technology reflected in the production relations is to clarify the fresh production mechanism and provide fresh production technology means and measures, thus turning into new relations of production.

#### 3.1.2. Capital Input

Capital input is the basis of national EG [36,37]. To some extent, national EG is partly due to the expansion of input in human capital and the scale of production, as well as the increase in social productivity. Once capital is invested, first on the supply side, an increase in output is driven by an increase in product size and productivity, leading to an increase in aggregate income. The increase in income will lead to an improvement in the productive capacity of savings, which will be converted into investment and thus lead to the growth of investment. In terms of aggregate demand, purchasing demand and productive capacity increase with income, which leads to an increase in investment funds. In this way, the mutual promotion of market supply and aggregate demand leads to a virtuous circle in the growth of the national economy.

#### 3.1.3. Human Capital

Due to the differences in human knowledge and technology, the labor element in economic activities also varies greatly, which affects the differences in productivity. Therefore, the development of the national economy must continue to increase the production of labor productivity and physical capital, but to a large extent, it also depends on social human capital, which is reflected in the human body of labor technology and product knowledge reserves [26,38]. Therefore, the investment in social human capital, such as education and training of social workers, will improve the basic quality of social workers, thereby increasing the reserve of social human capital, improving labor productivity, and promoting the growth of the national economy. Due to the diversified demand for workers and professionals for the development of the national economy, innovative scientific and technological talents, specialized professional talents, and skilled workers are required, and a certain level of technical education is also required.

#### 3.1.4. Improvement of C-D Production Function

In recent years, climate change and low-carbon transition have made scholars pay more attention to the key role of CED in EG [39,40,41,42,43,44,45,46,47,48]. As an independent factor of production, CED is applied to the extended C-D production function that includes energy factors.

China’s rural areas are still in the process of urbanization, which will not only promote a large number of the surplus agricultural labor force into city employment but also increase the accumulation of human resources and promote agricultural productivity improving further. In addition, as China has entered the technology-driven stage, the level of technological innovation capability and industrialization have had a significant impact on China’s EG. Meanwhile, since the country’s reform and opening-up, with the acceleration of China’s industrialization process, it has promoted the improvement of China’s industrialization level and further promoted the rapid growth of China’s economy and society. Therefore, in this paper, CED, urbanization level, scientific and technological innovation level, and industrialization level are introduced into the C-D production function as follows.


(1) $$Y_{\mathrm{it}}={\mathrm{AK}}_{\mathrm{it}}^{\alpha }L_{\mathrm{it}}^{\beta }{\mathrm{CE}}_{\mathrm{it}}^{\beta_{1}}{\mathrm{URB}}_{\mathrm{it}}^{\beta_{2}}{\mathrm{PAT}}_{\mathrm{it}}^{\beta_{3}}{\mathrm{lndu}}_{\mathrm{it}}^{\beta_{4}}$$

In the Formula (1): the subscript *i* = 1, …, 30 which represents each province and the subscript *t* = 2000, …, 2019 which represents the specific year. *Y*, *K*, *L*, *CE*, *URB*, *PAT*, and *Indu* represent the production level, capital stock, labor stock, clean energy development, urbanization level, technological innovation level, and industrialization level, respectively.

In order to reduce the absolute value of the data and facilitate the calculation, we adopt logarithmic processing for the variables involved in the Formula (1), so the form of the above production function is changed to Formula (2):

(2) $$\begin{array}{l} lnY_{it}=\beta_{0}+\beta_{1i}lnK_{it}+\beta_{2i}lnL_{it}+\beta_{3i}lnCE_{it}+\beta_{4i}lnURB_{it}+\beta_{5i}lnPAT_{it}\\ +\beta_{6i}lnlndu_{it}+\alpha_{i}+\nu_{t}+\varepsilon_{it} \end{array}$$

### 3.2. Establishment of Spatial Spillover Effect Model

According to SECM and the panel data model, the following basic model of spatial panel data is proposed. The standard panel data format is as shown in Formula (3):

(3) $$y_{it}=\beta_{0}+\beta_{it}x_{it}+\alpha_{it}$$

If the variable area is spatially autocorrelated, we can further build a spatial panel data model. The basic form of the spatial panel data model is as shown in Formula (4):

(4) $$y_{it}=\rho {W^{\prime }}_{i}y_{it}+\beta_{it}{x^{\prime }}_{it}+\alpha_{i}+\varepsilon_{it}$$

In Formula (4), *W_i_^’^* represents the *i*th row of the spatial weight matrix.

(5) $${W^{\prime }}_{i}y_{it}=\sum_{j=1}^{n}W_{ij}y_{jt}$$

*W_ij_* refers to the element of the spatial weight matrix *(i,j)*; *α_i_* represents the individual effect of region *i*th. If the correlation coefficient between *α_i_* and *x_it_’* is not 0, use the fixed effects model, otherwise use the random effects model. Therefore, the spatial panel data model can be extended to Formula (6).

(6) $$\begin{array}{c} y_{it}=\tau y_{i,t-1}+\rho {W^{\prime }}_{i}y_{it}+\beta_{it}{x^{\prime }}_{it}+dX_{t}\delta +\alpha_{i}+v_{t}+\varepsilon_{it}\\ \varepsilon_{it}=\lambda {m^{\prime }}_{i}\varepsilon_{t}+u_{it} \end{array}$$

In Formula (6), *y_i,t−1_* represents the first-order lag term of the explained variable; *dX_t_δ* represents the spatial lag of the explanatory variable; *v_t_* represents the time effect; *m_i_^’^* is the *i*th row of the spatial weight matrix of the disturbance term. Therefore, when *λ* = 0, it is modeled as an SDM; when *λ* = 0 and *δ* = 0, it is modeled as a spatial lag model (SLM); when *λ* = 0, *δ* = 0, and *τ* = 0, it is modeled as a spatial error model (SEM).

In the model, the spatial lag terms of the explained variables, key explanatory variables, and control variables are introduced, which are further extended to a spatial panel econometric model as shown in Formula (7):

(7) $$\begin{array}{c} lnY_{it}=\beta_{0}+\beta_{1i}lnK_{it}+\beta_{2i}lnL_{it}+\beta_{3i}lnCE_{it}+\beta_{4i}lnURB_{it}+\beta_{5i}lnPAT_{it}\\ +\beta_{6i}lnIndu_{it}+\theta_{1}WlnCE_{it}+\theta_{2}W\ln K_{it}+\theta_{3}W\ln L_{it}\\ +\theta_{4}W\ln URB_{it}+\theta_{5}W\ln PAT_{it}+\theta_{6}W\ln Ind_{it}+\alpha_{i}+\upsilon_{i}+\varepsilon_{it} \end{array}$$

In Formula (7), *ρ* is the elastic coefficient of the spatial lag term of the explained variable, *θ*_1_~*θ*_6_ is the elastic coefficient of the spatial lag term of the explanatory variable, and *W* is the spatial weight matrix. There are many methods that can be used to deal with methods involving spatial dependence and heterogeneity. In the analysis of economic geography, the more common method is to quantify the geographic information (such as latitude and longitude) of sample observation points by directly constructing a weight matrix, or to embed the economic information to form a nested weight matrix, and the matrix and traditional econometrics are combined to realize the analysis of spatial econometrics.

Let the sample size be *n*, denote *n_i_* as the observed value of the *i*th region, and denote *W_ij_* as the spatial relationship between the *i*th region and the *j*th region, then the spatial weight matrix *W* can be expressed as the following square matrix (8):

(8) $$W=\left(\begin{array}{ccc} w_{11} & \ldots & w_{1n}\\ \ldots & \ldots & \ldots \\ w_{n1} & \ldots & w_{nn} \end{array}\right)$$

Normally, if the spatial relationship between the *i*th region and itself is zero, then the principal diagonal element of the spatial weight matrix *w_ii_* = 0. At the same time, the spatial correlation of the two regions is independence of the order, i.e., the matrix value *w_ij_* = *w_ji_* for the spatial correlation between the *i*th region and the *j*th region. Considering that the weight matrix of distance space containing economic factors may have endogenous effects, the weight matrix of geographical distance space is constructed in this paper.

According to the first law of geography, the degree of connection between two points that are close to each other in the same geographic location is usually higher than that between two points that are far apart. The distance weight matrix is often used for distance measurement methods such as Euclidean distance, Manhattan distance, arc distance, etc. The more common method is to use the Euclidean distance as a standard, assuming the influence range (bandwidth) of the region and to construct the following weight parameter function (9):

(9) $$w_{ij}=\left\{\begin{array}{l} 0,d_{ij}\geq l_{\max}\cup d_{ij}\leq l_{\min}\\ \frac{1}{d_{ij}},l_{\min}<d_{ij}<l_{\max} \end{array}\right.$$

Formula (9) is often referred to as the inverse distance spatial weight matrix. Where *d_ij_* is the distance between the two regions and *l_min_* and *l_max_* are the lower and upper limits of the bandwidth, respectively. If *d_ij_* is within the bandwidth set, the weight value will take the inverse of the distance, otherwise it will take 0. This method requires artificially setting the upper and lower limits of the bandwidth, which has a certain degree of subjectivity. Philosophically, everything is universally connected, so some scholars set the lower limit of the bandwidth to 0 and the upper limit to positive infinity, i.e., no matter how far the distance is, it can be related; at this point, any *w_ij_* value is equal to $\frac{1}{d_{ij}}$.

### 3.3. Variable Description and Statistical Characteristics

The paper selected the annual panel statistical data of China’s 30 provinces in 2000–2019. Due to the serious lack of relevant data for Tibet, Hong Kong, Macao, and Taiwan, the sample of this paper does not cover these four regions. Table 1 shows the definition of the variables.

#### 3.3.1. Explained Variable

The explained variable is the output level (Y, unit: CNY), and the real GDP per capita of each province is utilized to reflect EG Determined by the ratio of actual GDP to the national resident population. These data come from statistical yearbooks of various provinces over the years.

#### 3.3.2. Explanatory Variables

According to the theoretical analysis above, the C-D production function containing the energy element is constructed. The seven explanatory variables are as follows:

(1) CED (unit: GW h). Because China has been confronted with the problem of reabsorption of clean energy sources, the total output of CEP in China is higher than the final consumption for reasons including technology and markets. Therefore, this paper uses the total output of CEP to evaluate CED. This indicator is a more accurate and comprehensive measure of CED in China. Data are taken from the electricity production in the primary energy in the energy balance sheet and the clean energy power generation published in the China Energy Statistical Yearbook.
(2) The number of labor force (L, unit: 10,000 people), measured from the item “*Employees by three industries*” published in the statistical yearbook of each province over the years.
(3) Capital stock (K, unit: 100 million CNY). According to the combined calculation of all enterprises and then according to the calculation result formula of the perpetual inventory method [49], the fixed capital stock can be calculated. The specific calculation formula is:
  (10) $$K_{it}=K_{i,t-1}(1-\delta_{it})+\frac{I_{it}}{P_{it}}$$
  In the Formula (10), *K_it_* and *K_i,t−1_* represent the capital stock of the *i*th province in year *t* and *t − 1*, respectively. *δ_it_*, *I_it_*, and *P_it_* represent the depreciation rate of fixed assets which are valued at 9.6%, gross fixed asset formation (i.e., new investment), and the fixed asset investment price index of province *i* in year t. The initial capital stock, i.e., the capital stock in the base period, is divided by 10% of the province’s total fixed asset formation in 2000. Thus, the capital stock of each province from 2000 to 2019 can be calculated with 2000 as the base period. The data are taken from the statistical yearbooks of various provinces over the years.
(4) Urbanization level (URB), as indicated by urbanized population as a percentage of the total population published in the provincial statistical almanacs over the years.
(5) The level of scientific and technological innovation (PAT, unit: piece), which is shown by the number of patents granted in each province published in the “*China Science and Technology Statistical Yearbook*” over the years.
(5) The level of industrialization (Indu), which is reflected by the percentage of industrial added value in gross national product released in provincial statistical yearbooks over the years.

#### 3.3.3. Variable Statistical Characteristics

Each variable is taken in a logarithm. The descriptive statistical results for each variable are presented in Table 2.

## 4. Empirical Analysis

### 4.1. Spatial Correlation Test

Spatial autocorrelation refers to the potential interdependence between observations of multiple variables within the same distribution area. Moran’s index I (Moran’s I) is the most important clustering test method, which can detect the similarity, dissimilarity, or spatial independence between adjacent areas in the whole research area. The calculation of Moran’s I is shown in Formula (11):

(11) $$I=\frac{n}{S_{0}}\frac{\sum_{i=1}^{n}\sum_{j=1}^{n}w_{ij}(x_{i}-\bar{x})(x_{j}-\bar{x})}{\sum_{i=1}^{n}{\left(x_{i}-\bar{x}\right)}^{2}}$$

where *x_i_* represents the observation value of the *i*th unit in the space and $\bar{x}$ represents the arithmetic mean of *x*. *w_ij_* is the spatial weight matrix, which describes the topological association of each spatial unit, and *S_0_* is the sum of all non-zero elements in the spatial weight matrix *w_ij_*.

When the Moran’s *I* value is in (−1,0), it is expressed as a negative spatial correlation, showing the characteristics of high- and low-value aggregation. The smaller the value range, the greater the spatial difference. If the Moran’s *I* value is in the range (0,1), it represents a positive spatial correlation, showing the characteristics of clustering of high values and high values, or clustering of low values and low values. The bigger the Moran’s *I* value, the greater the spatial difference. However, if the Moran’s *I* value is equal to zero, it means that the region is randomly distributed and has no spatial correlation.

Therefore, before investigating the spatial spillover effect of CED on EG, it is essential to first investigate the spatial autocorrelation and whether the spatial autocorrelation is significant. We will test for spatial autocorrelation using the geographic distance spatial weight matrix.

The spatial correlation coefficient between EG and CED from 2000 to 2019 was calculated based on the geographic distance spatial weight matrix. It can be seen from Table 3 that at the 1% significance level, the Moran’s *I* value of EG and CED are positive numbers. This means that there is a more obvious positive spatial correlation between them, and the effect of spatial agglomeration is more obvious. We can carry out a spatial econometric analysis.

**Figure 1:**
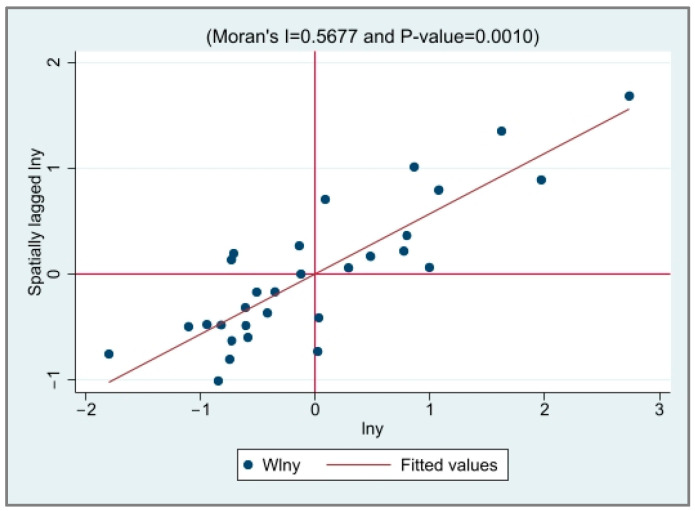
The scatter plot of the Moran index of EG in 2000.

**Figure 2:**
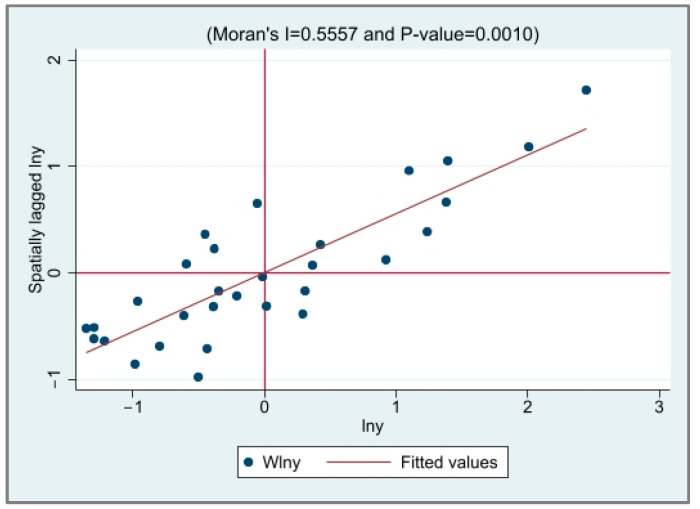
The scatter plot of the Moran index of EG in 2019.

**Figure 3:**
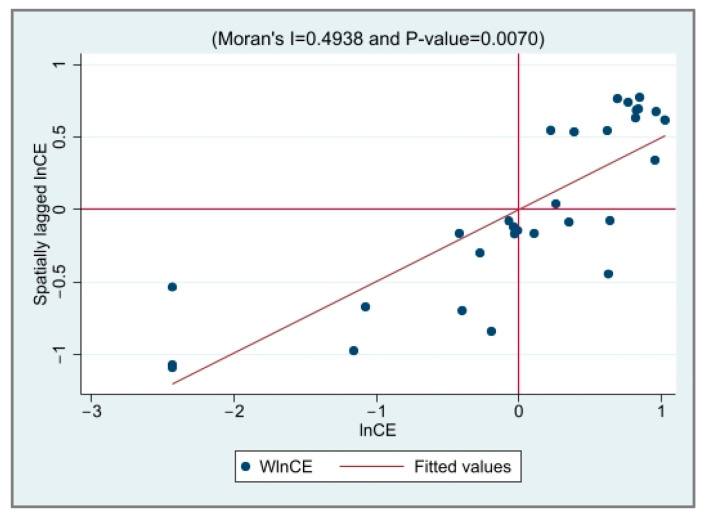
The scatter plot of the Moran index of CED in 2000.

**Figure 4:**
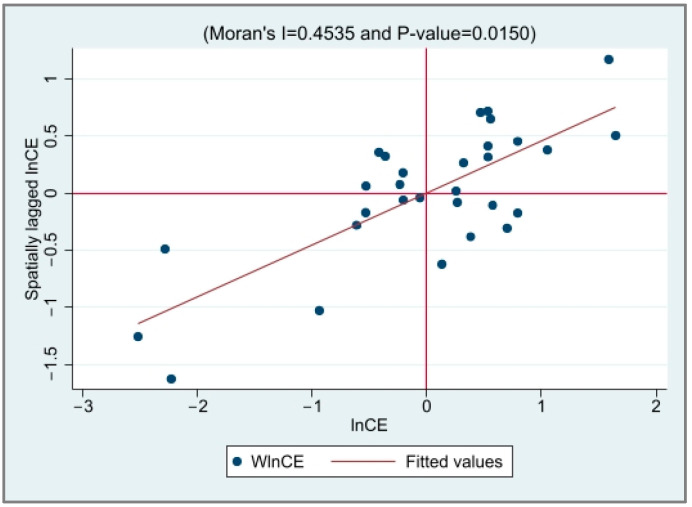
The scatter plot of the Moran index of CED in 2019.

The local Moran’s *I* value scatter plot of EG and CED was drawn to test the spatial correlation between the provinces, as shown in Figure 1, Figure 2, Figure 3 and Figure 4. It is not difficult to see from the local Moran’s index scatter plot that the EG and CED in most parts of China are located in the first and third quadrants, showing high–high agglomeration and low–low agglomeration, respectively, which further reflects the strong spatial positive correlation and spatial aggregation phenomenon.

### 4.2. Spatial Panel Model Selection

Relevant tests are needed to determine whether the SDM, the SEM, or the SLM should be selected. We first use LM (error) and Robust-LM (error) to detect spatial correlation and then use LM (Lag) and Robust-LM (Lag) to detect spatial lag. The test results are shown in Table 4.

As shown in Table 4, all of the test results are significant at the 1% level, indicating the presence of both spatial lag terms and spatial error terms in the model. Based on Elhorst’s conclusions, when there are both spatial error effects and spatial lag effects, both the LR test and the Wald test should be used simultaneously to select the correct SECM [50]. The test results show that since both the LR test and the Wald test pass the 1% significance test, the SDM cannot be degenerated into SEM or SLM, so this paper chooses the SDM for spatial analysis.

### 4.3. Regression Results of the Spatial Panel Model

According to Hausman’s test conclusion, if the *p*-value is about 0.001 and passes the 1% significance test, the null hypothesis should be rejected. After the second test to determine whether the time–space double fixed model should be selected as the optimal estimate, the *p* values were all 0.000 and the 1% significance test was passed. Therefore, the null hypothesis should be rejected. It is more feasible to use the time–space double fixed model for spatial econometric analysis while applying the SDM. The regression results are shown in Table 5.

The impact of CED on EG in the province is −0.003, but the coefficient is insignificant, indicating that the impact of CED on EG is not obvious. The impact of CED in neighboring provinces on EG in the province is 0.089, and the coefficient is significant at the 1% level. In the case where only the spatial proximity is taken into account, the CED in neighboring provinces can be effective in promoting the EG in the province.

### 4.4. Spillover Effect

The direct effect can be understood as a change in a given explanatory variable related to a space or unit of observation that has an intuitive impact on the area (explained variable) itself. Indirect effects (spillover effects) can be understood as an indirect form factor, i.e., a change in a given explanatory variable will not only directly influence the region itself but will also directly influence people or societies outside the region (explained variables). The SDM is Formula (12):

(12) $$\begin{array}{c} y=\rho Wy+X\beta +WX\theta +\varepsilon \\ \varepsilon ∼N(0,\sigma^{2}I_{n}) \end{array}$$

Therefore, the partial differential matrix of the explanatory variable *y* to the *k*th variable in the explanatory variables (*x_ik_*, *i* = 1, …, *N, i* represents the *i*th spatial observation unit) at time t is Formula (13):

(13) $$\left(\frac{\partial Y}{\partial x_{1k}}\cdots \frac{\partial Y}{\partial x_{Nk}}\right)=\left(\begin{array}{ccc} \frac{\partial y_{1}}{\partial x_{1k}} & \ldots & \frac{\partial y_{1}}{\partial x_{Nk}}\\ \vdots & \ddots & \vdots \\ \frac{\partial y_{n}}{\partial x_{1k}} & \ldots & \frac{\partial y_{n}}{\partial x_{Nk}} \end{array}\right)={\left(I-\rho W\right)}^{-1}\left(\begin{array}{cccc} \beta_{k} & W_{12}\theta_{k} & \cdots & W_{1N}\theta_{k}\\ W_{21}\theta_{k} & \beta_{k} & \cdots & W_{2N}\theta_{k}\\ \vdots & \vdots & \ddots & \vdots \\ W_{N1}\theta_{k} & W_{N2}\theta_{k} & \cdots & \beta_{k} \end{array}\right)$$

The average of the elements on the rectangle line in the matrix Formula (13) is the direct effect, and the average of the sums of the rows or columns of the non-rectangular elements is the spillover effect. Table 6 shows the decomposition estimation results of the spatial effect of CED calculated based on the SDM.

The indirect effect coefficient of CED on EG is 0.210, which is significant at the 1% level. This shows that the spillover effect of the CED in the province to the surrounding areas is positive, which is conducive to the promotion of EG in neighboring provinces. First of all, as we noted earlier, CEP and related clean energy technologies, projects, and know-how have more radiating paths and are more powerful than clean energy consumption in terms of spatial spillovers. Second, CED in the province will also have a demonstration effect on the surrounding area. CED in the province will play a benchmark role in reducing carbon dioxide emissions in the surrounding areas, which may force surrounding governments to increase environmental regulations to meet the environmental demands of residents in the region, thereby affecting CED and EG. Third, as the natural environment improves, these neighboring provinces will become more attractive to high-tech talent, and the capacity for innovation in science and technology will be enhanced. This will also have a positive impact on their EG. Moreover, once CED in the province is recognized and supported by the central government, it leads to intense competition among surrounding governments and is also an important pathway for CED to spill over to surrounding provinces.

### 4.5. Robustness Test

This paper intends to use the method of replacing the expression index of control variables and adding control variables to test the robustness of the spatial spillover effect of CED on EG. The specific method is described below.

Since China’s reform and opening-up in 1978, with the accelerating pace of economic globalization, the relationship between promoting China’s economic opening-up to the outside world and EG has become increasingly close. Therefore, the robustness test of this paper considers adding the degree of openness (trade) as a control variable. It is measured by the share of total imports and exports in the gross national product. The data are taken from the statistical almanacs of the provinces.

In addition, in the above model of the study of the spatial spillover effect of CED on EG, the level of industrialization uses the industrial added value in the gross national product, as the data are taken from the provincial statistical almanacs over the years. In fact, industrialization as a dynamic process of EG is seen more as the essence of continuous adjustment, optimization, and upgrading of the industrial structure, which is the process of shifting EG from primary to secondary and tertiary industries. Therefore, the “share of the output value of non-agricultural industry”, i.e., the share of the output value of the secondary and tertiary industries in the GDP, is used to reflect the level of industrialization.

Table 7 presents the results of the robustness test. The significance and the signs of the influence coefficients of the main explanatory variables are consistent with the previous estimation results in Table 5. This shows that the impact of CED on EG has not changed by changing the expression indicators of the control variables and adding new control variables, so the results of the study on the spatial effect of CED on EG are robust.

### 4.6. Endogeneity Test

As spatial spillovers from CED have an impact on regional EG, differences in regional EG may in turn influence the magnitude and direction of spatial spillovers from CED. Therefore, the empirical testing methodology in this paper is subject to certain endogeneity problems. To further overcome the potential endogeneity problem of the model in this paper, we employ the GMM approach of the spatial SAR model, which can both eliminate the influence of lagged variables and solve the endogeneity problem of the model itself. Following Bai et al. (2017), we chose W × lnCED as the instrumental variable. The *p*-value of the Sargan test estimate is greater than 0.1, thus accepting the original hypothesis that the instrumental variable is valid. Table 8 presents the results of the endogeneity test, which shows that the main conclusions are consistent with the previously estimated results presented in Table 5. Therefore, after taking endogeneity into account, we believe that CED still has a significant spatial spillover effect on regional EG.

## 5. Discussion

From the above analysis, the main conclusion is that CED has no significant impact on the EG of China’s 30 provincial administrative units. However, it does have a spatial spillover effect on the EG of surrounding provinces. This finding is partially consistent with Bai et al. (2020), who showed that the spatial spillover effect is larger than the direct effect. It should be noted that the study in this paper is broader in scope, covering 30 provincial administrative units across China instead of 21 development regions. Furthermore, while a number of studies choose the clean energy consumption ratio as the core explanatory variable to measure the degree of CED, this paper uses the total output of the CEP to evaluate CED, taking into account the issue of absorption. In our view, this indicator is a more accurate and comprehensive measure of CED in China. This may also be one of the reasons why our results differ from those of Bai et al. (2020). For reasons including technology and markets, total CEP in China is greater than final consumption.

While this study provides some new insights, it also has limitations. For example, we only used a linear model to test the spatial spillover effect, and our research did not take into account the absorption rate of CEP in different provincial administrative units, and these research gaps need to be addressed in future studies.

## 6. Conclusions and Policy Recommendations

### 6.1. Conclusions

Based on panel data from 30 provincial administrative units in 2000–2019, this paper investigates the spatial spillover effect of CED on EG from the supply side using the SECM model in China. The main conclusions are as follows: 

First, a province’s CDP does not have a significant impact on its own EG. In our opinion, the possible reasons for this may be that the CDP in some areas may not have been fully absorbed in some periods. According to the National Energy Administration of China, the annual wind power abandoned in 2016 amounted to 49.7 billion kWh, four times more than in 2014. The total amount of wind abandoned in Gansu, Xinjiang, Inner Mongolia, Jilin, and Heilongjiang from 2014 to 2016 was nearly 80 billion kWh, equivalent to the annual electricity consumption of Tianjin in 2015. The wind abandonment rate in Gansu Province increased from 11% in 2014 to 43% in 2016, which clearly hampers EG in the provinces where CEP is produced and supplied.

Another possible reason is that, although the western region supplies the vast majority of CEP, the production and R&D of key equipment for clean energy projects ARE located in the eastern region, and investment in clean energy projects in the west has very limited local economic stimulus.

Second, the impact coefficient of CED in surrounding provinces on the EG of the province is 0.073, which shows that CED has a positive spatial spillover effect on the EG of surrounding regions. Types of impacts include, but are not limited to: the technology spillover effect, the demonstration effect, the competitive effect, the environmental effect, etc.

We believe that the spatial effect involving environmental improvements is an important pathway that cannot be ignored. As the IEP increases, the structure of energy production in a province is optimized; it can also reduce air pollution in the surrounding provinces and improve livability, thus favoring the clustering of talent and industry and ultimately having a positive impact on EG. For example, in recent years, Hebei Province has continued to utilize clean energy sources such as wind and solar power, and the proportion of renewable energy installations in total electricity installations has increased to 52.9%. This will undoubtedly benefit the EG of the neighboring city of Beijing, which was once plagued by air pollution. 

### 6.2. Policy Recommendations

The empirical conclusions contain the following policy implications.

(1) Since our research finds no evidence that CED can promote EG, local governments may not be sufficiently motivated to promote CED in their region. Therefore, it is necessary to introduce and improve clean energy support policies from the central government. First of all, the government should strengthen fiscal and tax policy support for clean energy, optimize subsidies for clean energy projects and products, improve the fully guaranteed purchase system for clean energy, and strengthen support for preferential tax policies for clean energy projects and products. Second, the central government should strengthen the land supply for clean energy. The central government should formulate a spatial layout plan for clean energy to ensure the demand for land for CED. Third, the government must improve the green financing system for clean energy and further increase support for clean energy projects through green bonds and green loans.
(2) Our empirical research shows that the CED in one region has a positive spillover effect on neighboring regions. The demonstration effect and competition effect are important mechanisms to achieve the spillover effect, so the Chinese government should set up some clean energy demonstration bases and establish a number of high-level clean energy projects. For example, the national clean energy microgrid, the “photovoltaic+” cooperation model, the complementarity of water, wind, and light, and integrated clean energy system solutions, etc.
(3) In addition, the spillover effect of CED from one region to neighboring regions is also realized through technology spillover, the exchange of professional personnel, and other approaches. Therefore, local governments in different provinces should implement various kinds of communication mechanisms and break down information and human capital barriers. Furthermore, local governments should effectively coordinate policies to avoid inefficient use of resources and carry out comprehensive cooperation in CED to promote coordinated development among provinces.

## Figures and Tables

**Table 1:** Definition of the variables.

Variable	Unit	Definition
***Explained variable***		
*Y*	CNY	The actual per capita GDP of each province
***Explanatory variables***		
*CED*	GW·h	The total output of CEP
*K*	100 million CNY	Capital stock
*L*	10,000 people	The number of labor force
*URB*	%	Urbanization level
*PAT*	piece	The level of scientific and technological innovation, as reflected in the number of patents granted
*Indu*	%	The level of industrialization

**Table 2:** Statistical description of the main variables.

Variable	Measured Value	Mean	Standard Deviation	Minimum	Maximum
*lnY*	600	9.91	0.74	7.96	11.75
*lnCED*	600	4.33	2.55	−4.60	8.14
*lnK*	600	9.92	0.98	7.36	12.05
*lnL*	600	7.57	0.80	5.62	8.87
*lnURB*	600	3.84	0.36	2.67	4.50
*lnPAT*	600	8.87	1.76	3.47	13.18
*lnIndu*	600	3.47	0.57	0.45	5.40

**Table 3:** Moran’s *I* value of EG and CED for 2000–2019.

Year	lnY	lnCED	Year	lnY	lnCED
2000	0.568 ***	0.494 ***	2010	0.571 ***	0.527 ***
2001	0.568 ***	0.522 ***	2011	0.563 ***	0.489 ***
2002	0.569 ***	0.511 ***	2012	0.557 ***	0.507 ***
2003	0.574 ***	0.468 ***	2013	0.551 ***	0.509 ***
2004	0.578 ***	0.513 ***	2014	0.544 ***	0.527 ***
2005	0.582 ***	0.528 ***	2015	0.541 ***	0.546 ***
2006	0.586 ***	0.526 ***	2016	0.541 ***	0.505 ***
2007	0.583 ***	0.548 ***	2017	0.543 ***	0.496 ***
2008	0.580 ***	0.599 ***	2018	0.545 ***	0.483 ***
2009	0.575 ***	0.599 ***	2019	0.557 ***	0.453 ***

Notes: ***, **, and * represent statistical significance at 1%, 5%, and 10% levels, respectively.

**Table 4:** Test results of LM, LR, and Wald.

Tests	Statistics	*p* Value
Lagrange multiplier (error)	29.103	0.000 ***
Robust Lagrange multiplier (error)	30.125	0.000 ***
Lagrange multiplier (lag)	19.017	0.000 ***
Robust Lagrange multiplier (lag)	20.039	0.000 ***
LR lag	33.19	0.000 ***
LR error	42.06	0.000 ***
Wald lag	31.48	0.000 ***
Wald error	37.65	0.000 ***

Notes: ***, **, and * represent statistical significance at 1%, 5%, and 10% levels, respectively.

**Table 5:** Spatial regression results of the time–space double fixed model of SDM.

Variable	Coefficient	Z Value
*lnCED*	−0.003	−1.14
*lnK*	0.231 ***	13.31
*lnL*	0.297 ***	−9.46
*lnPAT*	0.036 ***	6.52
*lnURB*	0.099 ***	5.80
*lnIndu*	0.051 ***	4.19
*W × lnCED*	0.089 ***	5.43
*W × lnK*	−0.118	−1.58
*W × lnL*	1.663 ***	8.52
*W × lnPAT*	−0.146 ***	−4.89
*W × lnURB*	0.075	1.06
*W × lnIndu*	−0.008	0.19
*Ρ*	0.581 ***	9.06
*Sigma^2^*	0.003 ***	17.22
*Loglikelihood*	883.68	/
*R^2^*	0.6468	/

Notes: ***, **, and * represent statistical significance at 1%, 5%, and 10% levels, respectively.

**Table 6:** The decomposition estimation results of the spatial effect of CED.

Variable	Direct Effect	Indirect Effect
*lnCED*	0.002 (0.56)	0.210 *** (3.83)
*lnK*	0.230 *** (13.21)	0.020 (0.12)
*lnL*	−0.218 *** (−6.37)	3.570 *** (6.17)
*lnPAT*	0.029 *** (5.02)	−0.295 *** (−4.01)
*lnURB*	0.106 ** (6.15)	0.305 ** (2.33)
*lnIndu*	0.053 *** (4.23)	0.042 (0.45)

Notes: ***, **, and * represent statistical significance at 1%, 5%, and 10% levels, respectively.

**Table 7:** The robustness test results.

Variable	Coefficient	Z Value
*lnCED*	−0.002	−0.98
*lnK*	0.242 ***	13.93
*lnL*	−0.297 ***	−9.13
*lnPAT*	0.034 ***	6.10
*lnURB*	0.100 ***	5.75
*lnIndu*	0.114 ***	3.44
*lnTrade*	0.022 *	2.30
*W × lnCED*	0.087 ***	5.36
*W × lnK*	−0.123	−1.58
*W × lnL*	1.68 ***	7.91
*W × lnPAT*	−0.150 ***	−4.67
*W × lnURB*	0.043	0.56
*W × lnIndu*	0.625 *	1.89
*W × lnTrade*	0.008	0.23
*Ρ*	0.592 ***	10.39
*Sigma^2^*	0.003 ***	17.23
*Loglikelihood*	883.75	/
*R^2^*	0.6745	/

Notes: ***, **, and * represent statistical significance at 1%, 5%, and 10% levels, respectively.

**Table 8:** The endogeneity test results.

Variable	Coefficient	T Value
*lnEG(−1)*	0.915 ***	51.010
*lnCED*	0.005 **	2.000
*W*_*lnEG*	0.077 **	2.39
*lnK*	−0.052 ***	−2.83
*lnL*	0.059 ***	2.77
*lnPAT*	0.005 **	2.17
*lnURB*	0.078 ***	4.75
*lnIndu*	0.141 ***	2.59
**Test statistic**	**Value**	***p*-Value**
*Rho*	0.077	0.017
*Sargan test*	29.239	1
*Wald test*	3,222,188	0.000

Notes: ***, **, and * represent statistical significance at 1%, 5%, and 10% levels, respectively.

## Appendix A

**Table A1:** The abbreviations of key terms.

Key Term	Abbreviations	Key Term	Abbreviations
Clean energy development	CED	Research and development	R&D
Clean energy production	CEP	Spatial Econometric Model	SECM
Clean energy industry	CEI	Spatial Durbin Model	SDM
Cobb–Douglas	C-D	Spatial Error Model	SEM
Economic growth	EG	Spatial Lag Model	SLM
Moran’s index I	Moran’s I		
